# Implementation of the Coordinated Transitional Care (CTraC) Program in VA: Overview and Lessons Learned

**DOI:** 10.1093/geroni/igaf122.482

**Published:** 2025-12-31

**Authors:** Caroline Madrigal, Jane Driver

**Affiliations:** VA Boston Healthcare System, Boston, Massachusetts, United States; VA Boston Healthcare System, Brockton, Massachusetts, United States

## Abstract

The Coordinated Transitional Care (CTraC) program is an evidence-based, nurse-led program designed to meet VA’s need for transitional care that can serve a geographically dispersed population. The goal of CTraC is to reduce negative post-hospital outcomes and improve Veteran/family empowerment during the early post-hospitalization period. The program is led by a registered nurse case manager who identifies and enrolls Veterans at high risk for readmission—the nurse participates in multidisciplinary discharge planning, visits eligible Veterans while inpatient, and completes post-discharge telehealth follow-up within 48-72 hours and weekly telehealth follow-up for 30 days. CTraC does not duplicate or replace existing care; it fills the gap between inpatient and outpatient services, and provides intensive symptom assessment, medication reconciliation, and case management. By targeting only patients at increased risk of readmission, CTraC focuses resources on Veterans who need it most. CTraC protocol’s feasibility and acceptability has been demonstrated in research and by its sustainment in clinical practice for over ten years. Since its inception, the program has served over 10,000 Veterans and consistently demonstrates a significant reduction in hospital readmissions, identification and reconciliation of medication discrepancies, and care coordination to needed services/supports. All of which has translated to substantial cost savings across the 15 VA sites where it operates. Important next steps toward dissemination and sustainment will be described in this presentation including ongoing program evaluation, standardization efforts across the enterprise, identification of sites for future dissemination, and development of the program’s business case to encourage long-term sustainment.

